# Inflammation mediates the association between the Prognosis Nutrition Index (PNI) and mortality in acute pancreatitis: evidence from international cohorts

**DOI:** 10.3389/fimmu.2025.1693888

**Published:** 2026-01-14

**Authors:** Lingwei Huang, Dan Xiao, Lingjie Yan, Juan Liao, Meimei Yang, Fei He

**Affiliations:** 1 Department of Geriatrics, The Affiliated Yongchuan Hospital of Chongqing Medical University, Yongchuan, Chongqing, China; 2 Chongqing Municipality Clinical Research Center for Geriatric Diseases, Yongchuan Hospital, Chongqing Medical University, Yongchuan, Chongqing, China; 3 Department of Internal Medicine, Sichuan University Hospital of Sichuan University, Chengdu, Sichuan, China; 4 Information Center, The Affiliated Yongchuan Hospital of Chongqing Medical University, Yongchuan, Chongqing, China; 5 Central Laboratory, The Affiliated Yongchuan Hospital of Chongqing Medical University, Yongchuan, Chongqing, China; 6 Department of Clinical Pharmacy, West China Hospital of Sichuan University, Chengdu, Sichuan, China

**Keywords:** acute pancreatitis, mediation analysis, mortality, prognostic nutritional index, systemic inflammatory response syndrome

## Abstract

**Background:**

The prognostic nutritional index (PNI), reflecting nutritional and immune-inflammatory status, has been linked to outcomes across a range of disorders. Its prognostic value in acute pancreatitis (AP) and the potential mediating role of systemic inflammatory response syndrome (SIRS) remain unclear.

**Objective:**

To investigate whether systemic inflammatory response syndrome (SIRS) mediates the linkage between the prognostic nutritional index (PNI) and risk of death from any cause during hospitalization, as well as short- and long-term outcomes, in patients with acute pancreatitis (AP).

**Methods:**

This was an international retrospective cohort study utilizing the U.S. MIMIC-IV dataset (v2.2) and a hospital-based dataset in China, encompassing 2,574 patients diagnosed with AP (MIMIC cohort: 941; Chinese cohort: 1,633). Analyses included multivariable Cox regression, receiver operating characteristic (ROC) curves, Kaplan–Meier survival curves, restricted cubic spline (RCS) modeling, subgroup analysis, and mediation analysis.

**Results:**

After adjustment for age, sex, comorbidities, and BISAP scores, PNI remained to serve as a standalone protective factor among AP patients (all HR < 1, P < 0.05). PNI exhibited strong predictive performance, particularly for long-term mortality. The low PNI exhibited markedly greater mortality compared with those with high PNI at all time points (log-rank P < 0.01). PNI demonstrated a significant nonlinear negative correlation with mortality, with more pronounced protective effects in certain subgroups. SIRS partially mediated the PNI–mortality association.

**Conclusion:**

PNI is an independent determinant of mortality in AP, offering complementary prognostic value to BISAP. SIRS partially mediates this association but is not the primary pathway.

## 1 Introduction

Acute pancreatitis (AP) represents a frequent and potentially fatal gastrointestinal emergency characterized by a steadily increasing global incidence (1–4). Although most patients present with mild, self-limiting disease, approximately 15–20% develop moderate-to-severe or severe acute pancreatitis (SAP), which is often accompanied by persistent systemic inflammatory response syndrome (SIRS), organ dysfunction, and a mortality rate as high as 20–30% (5–7). Therefore, early and accurate identification of high-risk patients is crucial for facilitating risk-stratified management, optimizing resource allocation, and improving clinical outcomes (8).

In current clinical practice, severity assessment is commonly performed using tools such as the BISAP score, APACHE-II, and Ranson score (9–11). However, these scoring systems may be limited in clinical application due to their complex calculations or suboptimal specificity and sensitivity (11, 12). Growing evidence suggests that patients’ nutritional and immune status are critical determinants in the onset and progression of AP (13–15). The Prognostic Nutritional Index (PNI), a simple measure reflecting both nutritional status and immune function (derived from serum albumin and lymphocyte counts), predicts postoperative outcomes, especially in patients with malignancies (16–19).

Despite this, there remains a paucity of large-scale investigations into the prognostic potential of PNI in AP, and its underlying mechanisms are not fully understood. We hypothesize that the systemic inflammatory response may mediate the relationship between nutritional-immune status and clinical outcomes. SIRS is the most prominent pathophysiological alteration in the early stages of AP and represents a central mechanism in the development of organ dysfunction (20, 21). Malnutrition and immunosuppression (low PNI) may impair the regulation of inflammation, thereby exacerbating the severity and duration of SIRS; conversely, an adequate nutritional-immune status (high PNI) may mitigate excessive inflammatory responses and ultimately improve prognosis (22, 23). However, this hypothesis has not yet been systematically validated in prospective or large-scale retrospective studies.

To address these knowledge gaps, the present study conducts an international cohort investigation extracted from the MIMIC-IV open-access critical care dataset in the United States and a large single-center cohort in China. The primary objectives are: (1) To assess the link of PNI with short- and long-term all-cause mortality in hospitalized AP patients; and (2) to investigate, for the first time, whether SIRS serves as an intermediary between PNI and all-cause mortality among AP patients. The findings of this study will provide new insights into the mechanisms through which PNI influences AP outcomes and provide an important theoretical foundation for targeted clinical interventions, such as nutritional support and immune-inflammatory regulation.

## 2 Methods

### 2.1 Research design and data sources

This retrospective international cohort investigation used data from two independent databases. The MIMIC-IV database (v2.2) is a large, publicly available single-center intensive care database that contains detailed information on inpatients receiving care at Boston’s Beth Israel Deaconess Medical Center during the period 2008–2019 (24). The Chinese cohort database comprises clinical data from the Affiliated Yongchuan Hospital of Chongqing Medical University, covering the period from January 2018 to June 2024. The MIMIC database primarily includes ICU patients, whereas the Chinese cohort comprises a substantial number of inpatients from general wards. The study protocol was approved by the Ethics Review Committee of the Affiliated Yongchuan Hospital of Chongqing Medical University (Approval Number: 2025EC0030). All personal data were de-identified, with informed consent waived due to the retrospective study design.

### 2.2 Research population

Inclusion criteria were: (1) age ≥18 years; (2) diagnosis of acute pancreatitis within 24 hours of admission; and (3) availability of complete key clinical data within 24 hours of admission.

Exclusion criteria encompassed severe underlying conditions that could independently confer a high risk of mortality or profoundly interfere with nutritional status, inflammatory response, and outcome assessment. These conditions were specifically defined as:

End-stage renal disease: Requiring long-term maintenance dialysis (hemodialysis or peritoneal dialysis).

Active malignancies: Presence of any uncured, actively treated, or metastatic solid or hematological malignancy. (Localized skin cancers and cancers with a disease-free survival of >5 years were not excluded).

Severe liver dysfunction: Clinical diagnosis of liver failure, or cirrhosis with radiologically or clinically confirmed ascites.

**Figure 1:**
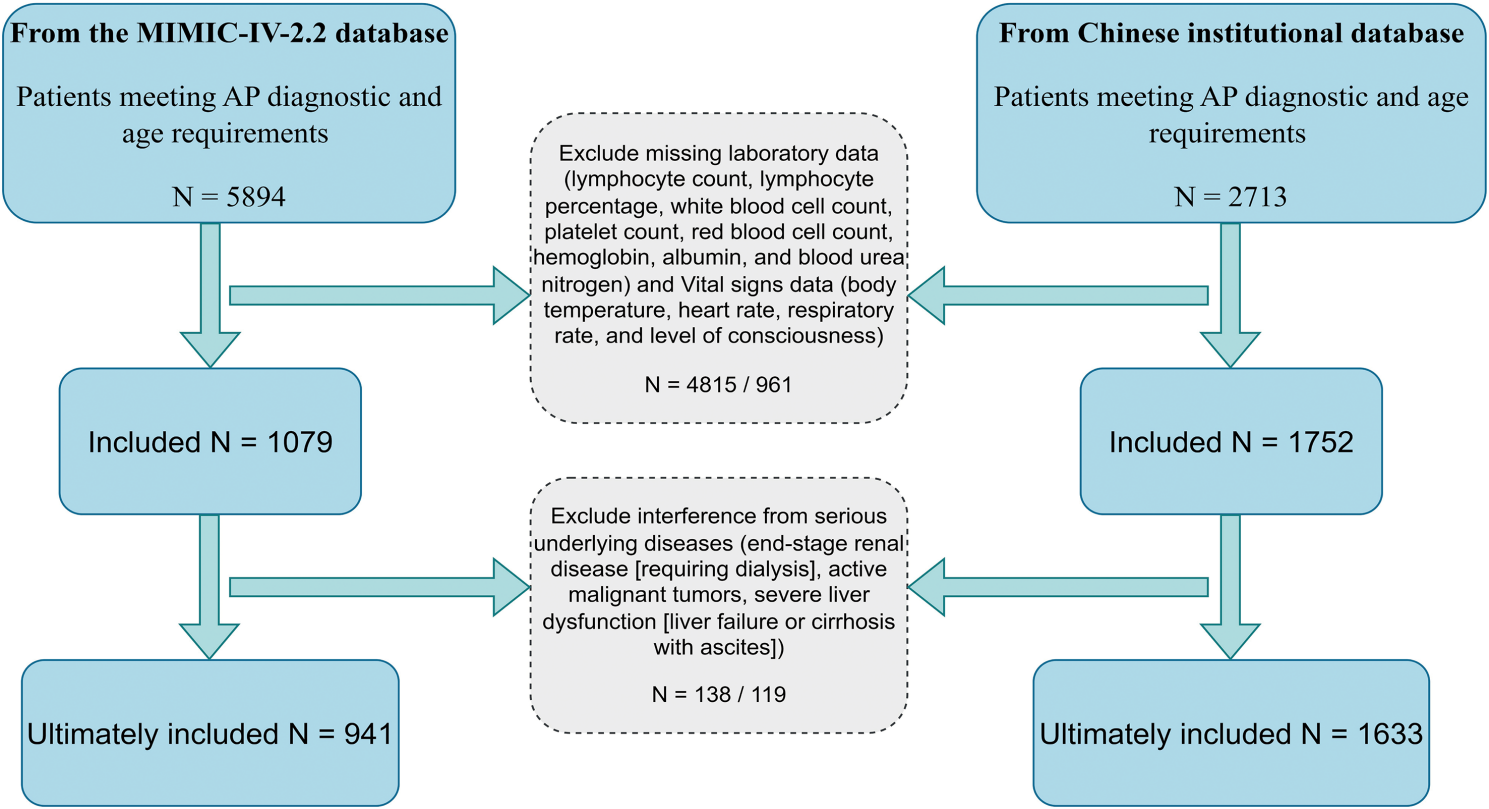
The patient inclusion flowchart. AP, Acute pancreatitis.

Patients with missing key covariates exceeding 20% were excluded. Multiple imputation was applied to variables with <20% missing values. The patient inclusion flowchart is shown in **Figure 1**. In the MIMIC-IV cohort, these conditions were primarily identified using International Classification of Diseases (ICD-9 and ICD-10) codes. Dialysis status was further verified against nursing plans and procedure records. In the Chinese cohort, identification was performed using a combination of ICD-10 codes and keyword searches in the structured electronic medical record system, with manual review of clinical notes conducted for ambiguous cases to ensure consistent application of the criteria across both cohorts.

### 2.3 Variable definition and data collection

All variables were extracted from the MIMIC-IV database and from the information center of the Affiliated Yongchuan Hospital of Chongqing Medical University via Structured Query Language (SQL) in PostgreSQL (24). To ensure harmonization of data across the two international cohorts, standardized definitions were applied for all key variables. Laboratory values were handled using standard international units. Comorbidities were defined using ICD-9 (MIMIC) and ICD-10 (MIMIC and Chinese cohort) codes, which were clinically reviewed for consistency. Baseline data were collected and included the following categories: (1) demographics (age, gender); (2) comorbidities (diabetes, hypertension, chronic obstructive pulmonary disease [COPD], heart failure); (3) laboratory indicators: all laboratory values (hemoglobin, lymphocyte count, platelet count, lymphocyte percentage, leucocyte count, albumin, and erythrocyte count) were obtained from the first measurement recorded within 24 hours of admission; (4) scoring systems: The Prognostic Nutritional Index (PNI) was calculated using the serum albumin (g/L) and lymphocyte count (×10^9^/L) from this first 24-hour measurement, defined as serum albumin + 5 × lymphocyte count. Similarly, the BISAP score was calculated identically in both cohorts using its five original components based on data from the first 24 hours of admission. This consistent timing for PNI and BISAP calculation ensured the comparability of their predictive performance in subsequent analyses. In contrast, the Systemic Inflammatory Response Syndrome (SIRS) score was assessed dynamically throughout the entire hospitalization period. The highest SIRS score recorded during the hospitalization was used to define the presence of SIRS (SIRS score ≥2) for the mediation analysis, thereby maximizing the assurance of temporal sequence among PNI, SIRS, and mortality outcomes in the mediation analysis. The SIRS score was defined according to established consensus criteria, based on ①Leukocyte count greater than 12 × 10^9^/L or less than 4 × 10^9^/L, or immature granulocytes more than 10%, ②Heart rate exceeding 90 beats per minute, ③Respiratory rate above 20 breaths per minute, or arterial partial pressure of carbon dioxide (PaCO_2_) below 32 mmHg, ④Body temperature exceeding 38 °C or falling below 36 °C (25). The BISAP score was calculated from five criteria (①Altered consciousness with a Glasgow Coma Scale score below 15, ②Blood urea nitrogen [BUN] exceeding 25 mg/dL [8.9 mmol/L], ③Signs of SIRS, ④ Age greater than 60 years, ⑤Presence of pleural effusion), each assigned 1 point (25); and (5) outcome measures, including all-cause mortality during hospitalization, as well as at 14, 30, 90, 180 days, and 1 year (3, 26). The distinct temporal assessment of these scores—baseline PNI and BISAP versus in-hospital SIRS—was designed to respect the temporal sequence required for the mediation analysis, positing PNI (baseline) influencing the subsequent development of SIRS (during hospitalization), which in turn affects the final mortality outcome. The BISAP score was employed as a pragmatic and well-validated tool for severity adjustment in both cohorts, ensuring consistency and data extractability in this retrospective study.

### 2.4 Statistical analysis

SPSS 26.0, MedCalc 22.0, and R 4.4.2 software were used. The MIMIC and Chinese cohorts were analyzed independently. Normally distributed continuous data, summarized as mean ± standard deviation (x ± s), were examined via the independent-samples t-test. Skewed distributions, summarized as median (IQR), were assessed via the Mann–Whitney U test. Categorical data, summarized as counts (percentages) [n (%)], were examined via the chi-square test. We calculated the standardized mean difference (SMD) for all variables presented in **Table 1** (27). The SMD was computed as the difference in means (or proportions for categorical variables) divided by the pooled standard deviation (27). Following common conventions, an absolute SMD of <0.1 was interpreted as a negligible difference, 0.1–0.2 as a small difference, and ≥0.2 as a meaningful difference in baseline characteristics between the groups (27). Cox regression models, including Model 1 (crude), Model 2 (age- and sex-adjusted), and Model 3 (additionally adjusted for diabetes, hypertension, heart failure, COPD, and BISAP score), were examined the effect of continuous PNI on mortality risk at different time points, with hazard ratios (HRs) and 95% confidence intervals (CIs) calculated. Three models were sequentially developed: Model 1 (unadjusted), Model 2 (adjusted for age and sex), and Model 3 (further adjusted for diabetes, hypertension, heart failure, COPD, and BISAP score). The variables for Model 3 were selected as key clinical confounders known to influence both nutritional-inflammation status and mortality, and were prioritized for their consistent availability and reliability across both international cohorts. To verify the consistency of the association between PNI and mortality across cohorts and to account for potential heterogeneity, a sensitivity analysis was performed using a shared frailty Cox proportional hazards model (a mixed-effects model) with cohort membership included as a random effect. Predictive performance of PNI and BISAP for mortality was examined via receiver operating characteristic (ROC) curves, with area under the curve (AUC) calculated and differences examined via the DeLong test. The optimal cutoff value, sensitivity, and specificity of PNI for predicting mortality were additionally determined. Based on the optimal cutoff for 30-day mortality, patients were categorized into low- and high-PNI groups. Kaplan–Meier estimates constructed survival curves with group comparisons via the log-rank test. Nonlinear effects of PNI on mortality were examined via restricted cubic splines (RCS) within the fully adjusted model. Subgroup analyses were stratified by sex, age (cutoff 60 years), comorbidities, and BISAP score (cutoff 3 points), with interaction tests used to evaluate effect modification. Mediation analysis was performed using the SPSS PROCESS macro (v4.3, Model 4) to evaluate whether SIRS mediates the relationship between PNI and mortality. This analysis involved: (1) a linear regression model with SIRS as the outcome, and (2) a Cox proportional hazards model with mortality as the outcome. Both models included PNI as the independent variable and were adjusted for age, sex, diabetes, hypertension, heart failure, COPD, and BISAP score. The significance of the indirect effect was tested using the bias-corrected bootstrap method with 5,000 resamples; a 95% confidence interval excluding zero indicated significant mediation. The proportional hazards assumption was met, and no significant PNI×SIRS interaction was found, supporting the model application. A two-sided P < 0.05 was considered statistically significant. The MIMIC and Chinese cohorts were analyzed both independently and in a pooled manner. To account for the potential clustering of patients within cohorts in the pooled analysis, a shared frailty Cox proportional hazards model (a mixed-effects model) with cohort as the random effect was employed as a sensitivity analysis to verify the consistency of the primary findings.

**Table 1:** Baseline data.

Characteristics	MIMIC						CHINA					
	Total	PNI ≤ 37.33	PNI>37.33	Unmatched SMD	Matched SMD	P	Total	PNI ≤ 46.00	PNI>46.00	Unmatched SMD	Matched SMD	P
Number of patients, n (%)	941	471 (50.10)	470 (49.90)				1633	820 (50.21)	813 (49.79)			
Demographic characteristics												
Age, IQR, year	56.00 (45.00, 69.00)	58.00 (47.00, 70.00)	55.00 (44.75, 66.00)	-0.1733	0.0971	0.005	52.00 (40.50, 65.00)	57.00 (47.00, 72.00)	48.00 (36.50, 56.00)	-0.7406	0.0217	<0.001
Gender, n (%)						0.034						<0.001
Male	509 (54.09)	271 (57.50)	238 (50.60)	-0.1380	-0.0336		914 (55.97)	409 (49.90)	505 (62.10)	0.2523	-0.0346	
Female	274 (45.91)	200 (42.50)	232 (49.40)				719 (44.03)	411 (50.10)	308 (37.90)			
Comorbidities												
COPD, n (%)	81 (8.61)	40 (8.50)	41 (8.70)	0.0082	0.0596	0.900	83 (5.08)	67 (8.20)	16 (2.00)	-0.4466	-0.1358	<0.001
Heart failure, n (%)	108 (11.48)	61 (13.00)	47 (10.00)	-0.0984	0.0280	0.156	118 (7.23)	87 (10.60)	31 (3.80)	-0.3549	-0.0438	<0.001
Diabetes, n (%)	235 (24.97)	130 (27.60)	105 (22.30)	-0.1263	0.0706	0.062	490 (30.01)	247 (30.10)	243 (29.90)	-0.0051	-0.0641	0.918
Hypertension , n (%)	478 (50.80)	246 (52.20)	232 (49.40)	-0.0574	0.0336	0.379	449 (27.50)	265 (32.30)	184 (22.60)	-0.2314	0.0100	<0.001
Laboratory indicators												
Leucocyte count, IQR, ×10^9^/L	9.90 (6.74, 14.50)	11.40 (7.54, 16.68)	8.71 (6.20, 12.18)	-0.4980	-0.0158	<0.001	10.10 (7.20, 13.50)	10.00 (7.10, 13.30)	10.20 (7.40, 13.60)	-0.0117	0.0499	0.570
Erythrocyte count, ×10^12^/L	3.67 ± 0.66	3.47 ± 0.63	3.87 ± 0.62	0.6398	0.0498	<0.001	4.40 (3.95, 4.84)	4.15 (3.74, 4.62)	4.57 (4.21, 4.99)	0.6598	-0.0036	<0.001
Platelet count, IQR, ×10^9^/L	204.00 (139.54, 290.83)	191.00 (125.33,292.33)	213.25 (151.50, 284.50)	-0.0106	-0.0705	0.042	188.00 (151.00, 240.00)	181.00 (138.25, 233.00)	196.00 (159.00, 242.00)	0.2101	0.0509	<0.001
Hemoglobin, g/L	110.66 ± 19.25	104.75 ± 18.96	116.58 ± 17.67	0.6697	0.0423	<0.001	134.00 (120.00, 149.00)	127.00 (113.00, 140.00)	141.00 (128.00, 153.00)	0.6947	-0.0001	<0.001
Score system												
BISAP score, IQR, point	1.00 (0.00, 2.00)	2.00 (1.00, 3.00)	0.00 (0.00, 1.00)	-0.8739	0.1192	<0.001	1.00 (0.00, 2.00)	1.00 (1.00, 2.00)	1.00 (0.00, 1.00)	-0.6010	0.0069	<0.001
SIRS score, IQR, point	1.00 (1.00, 3.00)	3.00 (1.00, 4.00)	1.00 (1.00, 1.00)	-0.8988	0.0633	<0.001	1.00 (0.00, 2.00)	1.00 (0.00, 3.00)	1.00 (0.00, 2.00)	-0.1865	0.0414	0.001
Ending												
In-hospital mortality, n (%)	72 (7.65)	56 (11.90)	16 (3.40)	-0.4679	-0.0232	<0.001	44 (2.69)	32 (3.90)	12 (1.50)	-0.2012	0.0869	0.002
14-day mortality, n (%)	42 (4.46)	31 (6.60)	11 (2.3)	-0.2805	-0.0001	0.002	44 (2.69)	34 (4.10)	10 (1.20)	-0.2646	0.0761	<0.001
30-day mortality, n (%)	68 (7.23)	54 (11.50)	14 (3.00)	-0.4992	-0.0247	<0.001	73 (4.47)	56 (6.80)	17 (2.10)	-0.3312	0.0879	<0.001
90-day mortality, n (%)	108 (11.48)	79 (16.80)	29 (6.20)	-0.4406	0.0349	<0.001	91 (5.57)	72 (8.80)	19 (2.30)	-0.4265	0.0833	<0.001
180-day mortality, n (%)	138 (14.67)	99 (21.00)	39 (8.30)	-0.4612	0.0457	<0.001	110 (6.74)	87 (10.60)	23 (2.80)	-0.4693	0.0885	<0.001
1-year mortality, n (%)	169 (17.96)	118 (25.10)	51 (10.90)	-0.4566	0.0405	<0.001	153 (9.37)	123 (15.00)	30 (3.70)	-0.5999	0.0556	<0.001
Length of hospitalization, IQR, day	7.00 (4.00, 14.50)	10.00 (6.00, 19.00)	5.00 (3.00, 9.00)	-0.4639	-0.0583	<0.001	7.00 (5.00, 11.00)	8.00 (6.00, 12.00)	7.00 (5.00, 10.00)	-0.2379	0.0037	<0.001

PNI, prognostic nutritional index; IQR, interquartile range; COPD, chronic obstructive pulmonary disease; BISAP, bedside index of severity in acute pancreatitis; SIRS, systemic inflammatory response syndrome; SMD, standardized mean differences. We have interpreted an absolute SMD < 0.1 as indicating a negligible difference between the low-PNI and high-PNI groups, an absolute SMD between 0.1 and 0.2 as a small difference, and an absolute SMD > 0.2 as a meaningful difference. Prior to matching, most variables exhibited SMDs greater than 0.2, indicating significant baseline differences between the two groups. Following propensity score matching, the SMD for all variables decreased to below 0.1, demonstrating that the matching successfully balanced baseline characteristics.

## 3 Results

### 3.1 Baseline characteristics

**Figure 2:**
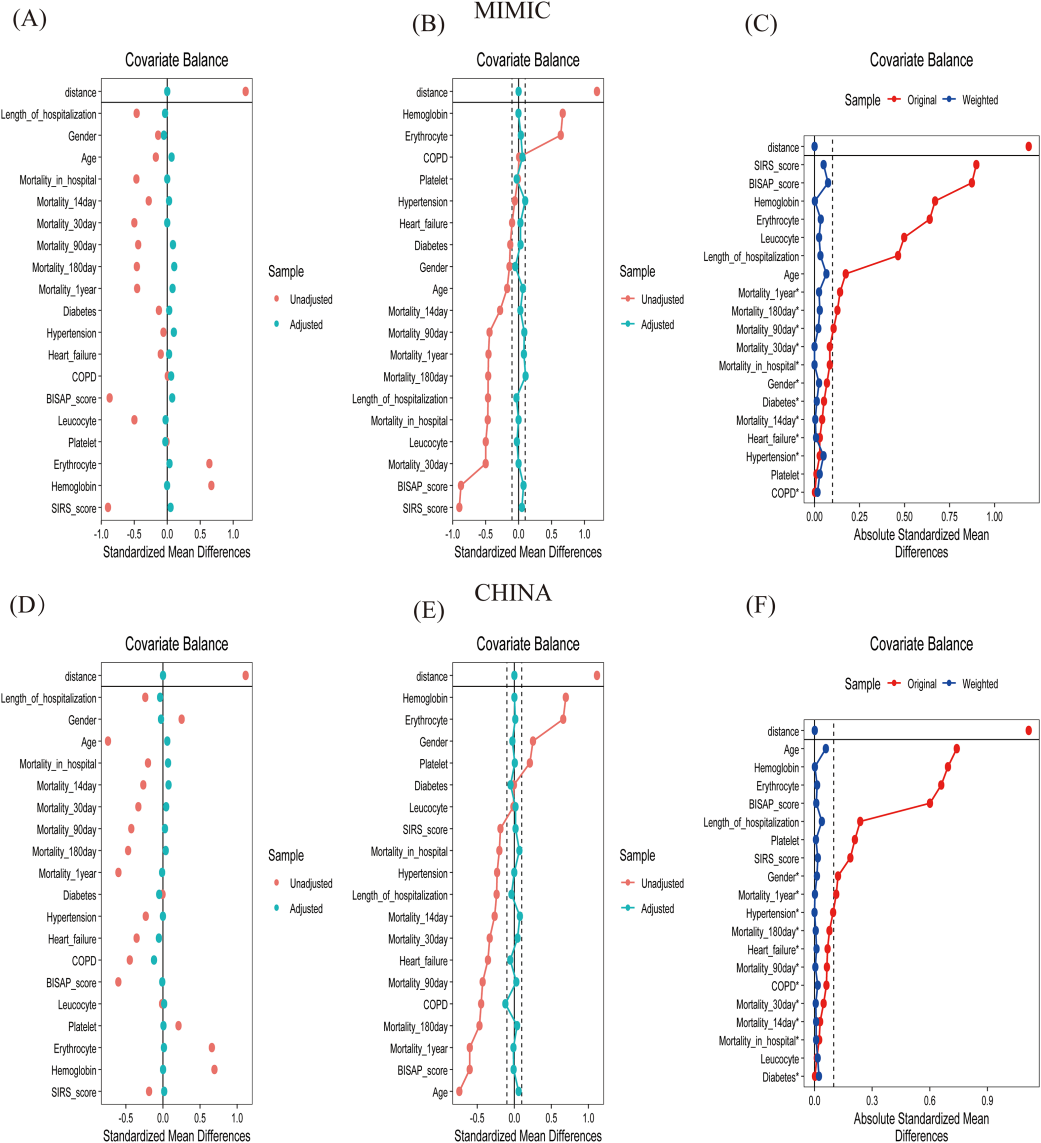
Standardized mean difference of baseline data. COPD, chronic obstructive pulmonary disease; BISAP, bedside index of severity in acute pancreatitis; SIRS, systemic inflammatory response syndrome. We have interpreted an absolute SMD < 0.1 as indicating a negligible difference between the low-PNI and high-PNI groups, an absolute SMD between 0.1 and 0.2 as a small difference, and an absolute SMD > 0.2 as a meaningful difference. Prior to matching, most variables exhibited SMDs greater than 0.2, indicating significant baseline differences between the two groups. Following propensity score matching, the SMD for all variables decreased to below 0.1, demonstrating that the matching successfully balanced baseline characteristics.

Overall, 2,574 AP patients comprised the study population, with 941 from the MIMIC cohort and 1,633 from the Chinese cohort. Based on the median PNI scores in each cohort (MIMIC: 37.33; China: 46.00), patients were categorized into low- and high-PNI groups, which differed significantly in baseline characteristics. As shown in **Table 1**, several consistent patterns were observed across cohorts, and the standardized mean differences (SMDs) provided in **Figure 2** further quantified the magnitude of these imbalances, reinforcing the clinical relevance of the PNI stratification. Older age characterized the low-PNI group, and the Chinese cohort had a higher burden of comorbidities, particularly COPD, heart failure, and hypertension. Nutritional indices (RBC count, hemoglobin, and platelets) were significantly lower in the low-PNI group, suggesting poorer nutritional status. In terms of severity, both BISAP and SIRS scores were significantly higher in the low-PNI group, indicating greater disease severity and heightened inflammatory response. Notably, all-cause death from admission to 1-year follow-up was greater in the low-PNI cohort, accompanied by extended hospital stays (all P < 0.01). Prior to matching, most variables exhibited SMDs greater than 0.2, indicating significant baseline differences between the two groups. Following propensity score matching, the SMD for all variables decreased to below 0.1, demonstrating that the matching successfully balanced baseline characteristics. These findings indicate that low PNI is strongly associated with adverse clinical outcomes.

### 3.2 Multivariable COX regression of PNI and mortality risk

As shown in **Table 2**, PNI independently conferred protection against mortality over both short- and long-term follow-up in both cohorts, and this association was consistent across populations. After controlling for age, sex, comorbid conditions, and BISAP score in Model 3, the protective association of PNI remained robust. In the MIMIC cohort, each 1-unit increase in PNI reduced the risk of 30-day (HR = 0.964, 95% CI: 0.931–0.998), 90-day (HR = 0.960, 95% CI: 0.934–0.986), and 1-year (HR = 0.951, 95% CI: 0.931–0.972) mortality. The Chinese cohort showed a stronger association, with each 1-unit increase in PNI significantly reducing 30-day (HR = 0.937, 95% CI: 0.904–0.972), 90-day (HR = 0.939, 95% CI: 0.909–0.970), and 1-year (HR = 0.940, 95% CI: 0.917–0.964) mortality. A sensitivity analysis using a shared frailty Cox model, which accounts for within-cohort clustering, confirmed the independent protective association of PNI with mortality across all time points (see **Supplementary Table S1**), supporting the robustness of the primary findings.

**Table 2:** COX regression analysis for continuous PNI.

Categories	Model 1 HR(95%CI)	Model 2 HR(95%CI)	Model 3 HR(95%CI)
MIMIC			
In-hospital mortality (PNI)	0.945(0.916,0.976)	0.948(0.918,0.979)	0.963(0.931,0.996)
14-day mortality (PNI)	0.965(0.928,1.004)	0.971(0.933,1.011)	0.990(0.953,1.027)
30-day mortality (PNI)	0.946(0.916,0.978)	0.950(0.919,0.981)	0.964(0.931,0.998)
90-day mortality (PNI)	0.945(0.921,0.970)	0.947(0.923,0.972)	0.960(0.934,0.986)
180-day mortality (PNI)	0.942(0.921,0.964)	0.943(0.921,0.965)	0.952(0.929,0.975)
1-year mortality (PNI)	0.944(0.925,0.963)	0.944(0.925,0.964)	0.951(0.931,0.972)
CHINA			
In-hospital mortality (PNI)	0.898(0.861,0.937)	0.898(0.859,0.938)	0.952(0.909,0.997)
14-day mortality (PNI)	0.890(0.853,0.928)	0.885(0.847,0.924)	0.933(0.891,0.977)
30-day mortality (PNI)	0.885(0.856,0.915)	0.886(0.857,0.917)	0.937(0.904,0.972)
90-day mortality (PNI)	0.884(0.858,0.911)	0.888(0.861,0.916)	0.939(0.909,0.970)
180-day mortality (PNI)	0.886(0.862,0.910)	0.896(0.871,0.922)	0.943(0.916,0.972)
1-year mortality (PNI)	0.887(0.866,0.907)	0.907(0.884,0.930)	0.940(0.917,0.964)

COX, proportional hazards model; HR, hazard ratio; CI, confidence interval; PNI, prognostic nutritional index; COPD, chronic obstructive pulmonary disease; BISAP, bedside index of severity in acute pancreatitis;

Model 1: No confounding factors were adjusted;

Model 2: Incorporated adjustments for Age and Gender;

Model 3: Incorporated adjustments for Age, Gender, COPD, Heart failure, Diabetes, Hypertension and BISAP score.

### 3.3 Predictive performance of PNI vs. BISAP scores

**Figure 3:**
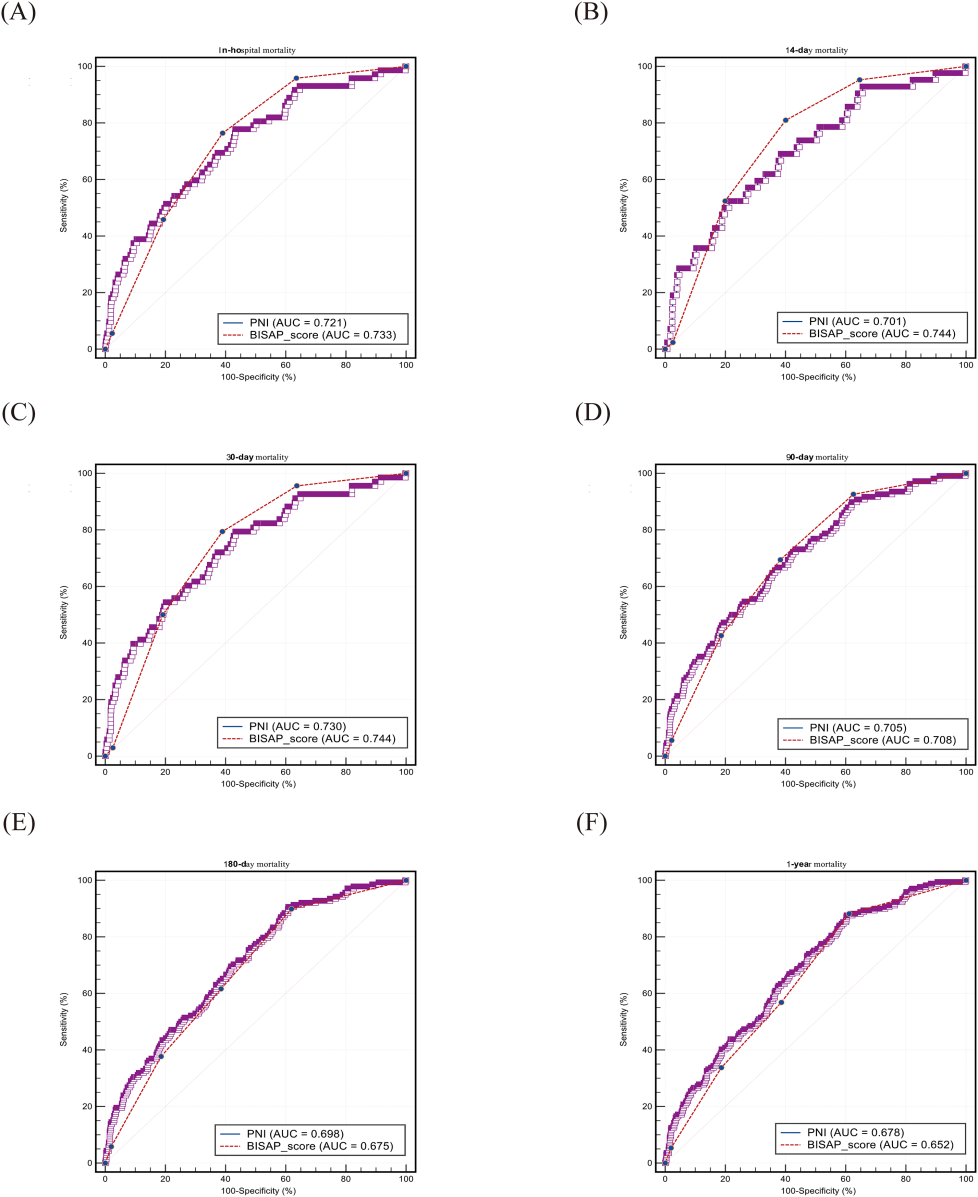
ROC curve from the MIMIC cohort. ROC, receiver operating characteristic curve; AUC, area under curve; PNI, prognostic nutritional index; BISAP, bedside index of severity in acute pancreatitis. The sensitivity and 100-specificity of the vertical and horizontal axes are expressed as percentages(%).

**Figure 4:**
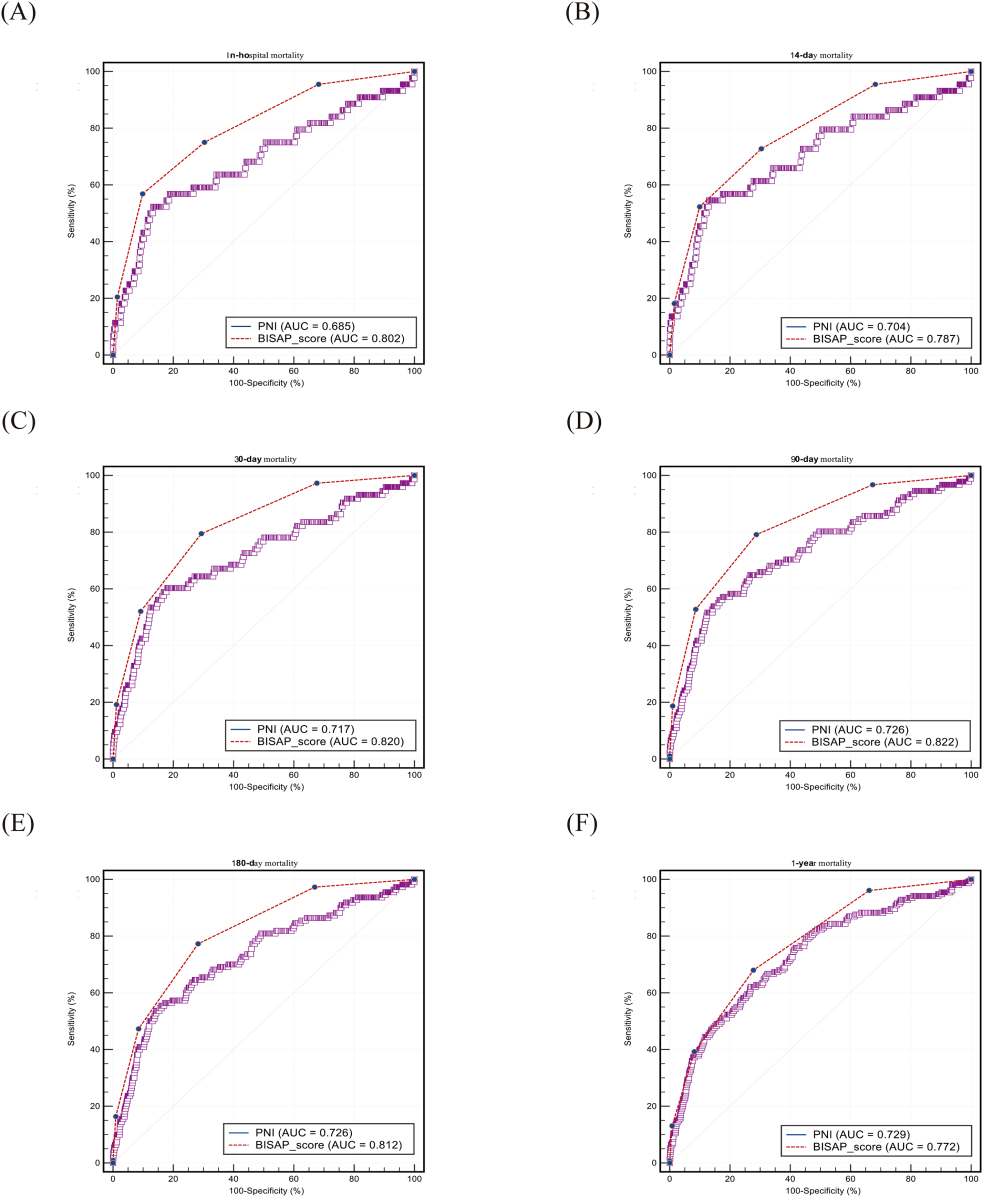
ROC curve from the Chinese cohort. ROC, receiver operating characteristic curve; AUC, area under curve; PNI, prognostic nutritional index; BISAP, bedside index of severity in acute pancreatitis. The sensitivity and 100-specificity of the vertical and horizontal axes are expressed as percentages(%).

ROC curve analysis (**Table 3**; **Figures 3**, **4**) showed that both PNI and BISAP scores were significant predictors of mortality (all AUCs, P < 0.001), although predictive performance varied by cohort. In the MIMIC cohort, the predictive accuracy of the two did not differ significantly, and PNI showed a trend of being no less effective than BISAP in predicting 180-day and 1-year mortality (AUC: 0.698 vs 0.675; 0.678 vs 0.652). In the Chinese cohort, BISAP demonstrated significantly superior predictive ability for short-term mortality (e.g., 30-day mortality, AUC: 0.820 vs. 0.717, DeLong P = 0.006). Optimal cutoff values for PNI were 31.4–40.2 (MIMIC) and 38.6–42.6 (China). Overall, PNI emerged as an effective predictor of mortality, especially for long-term outcomes.

**Table 3:** ROC curve analysis of PNI and BISAP score.

Variables	AUC value	95% CI	SE	Youden Index	Optimal cut-off value	Sensitivity (%)	Specificity (%)	Z value	P value	DeLong test P
MIMIC										
In-hospital mortality										0.7421
PNI	0.721	0.691 to 0.749	0.0319	0.3451	36.66	77.78	56.73	6.922	<0.0001	
BISAP score	0.733	0.703 to 0.761	0.0251	0.3738	1	76.39	60.99	9.271	<0.0001	
14-day mortality										0.3596
PNI	0.701	0.670 to 0.730	0.0427	0.3114	31.46	52.38	78.75	4.708	<0.0001	
BISAP score	0.744	0.715 to 0.772	0.0317	0.4091	1	80.95	59.96	7.684	<0.0001	
30-day mortality										0.7174
PNI	0.730	0.701 to 0.758	0.0329	0.3611	36.66	79.41	56.70	7.007	<0.0001	
BISAP score	0.744	0.715 to 0.772	0.0253	0.4047	1	79.41	61.05	9.641	<0.0001	
90-day mortality										0.9287
PNI	0.705	0.675 to 0.734	0.0263	0.2981	36.78	73.15	56.66	7.790	<0.0001	
BISAP score	0.708	0.677 to 0.737	0.0233	0.3115	1	69.44	61.70	8.922	<0.0001	
180-day mortality										0.4005
PNI	0.698	0.668 to 0.727	0.0231	0.2968	40.21	90.58	39.10	8.569	<0.0001	
BISAP score	0.675	0.644 to 0.704	0.0223	0.2796	0	89.86	38.11	7.826	<0.0001	
1-year mortality										0.3336
PNI	0.678	0.647 to 0.708	0.0218	0.2721	40.21	87.57	39.64	8.137	<0.0001	
BISAP score	0.652	0.621 to 0.683	0.0210	0.2703	0	88.17	38.86	7.251	<0.0001	
CHINA										
In-hospital mortality										0.0186
PNI	0.685	0.661 to 0.707	0.0490	0.3893	38.65	52.27	86.66	3.765	0.0002	
BISAP score	0.802	0.782 to 0.822	0.0349	0.4700	2	56.82	90.18	8.674	<0.0001	
14-day mortality										0.0838
PNI	0.704	0.681 to 0.726	0.0476	0.4146	38.55	54.55	86.91	4.286	<0.0001	
BISAP score	0.787	0.767 to 0.807	0.0350	0.4233	1	72.73	69.60	8.203	<0.0001	
30-day mortality										0.0064
PNI	0.717	0.694 to 0.739	0.0359	0.4226	40.05	60.27	81.99	6.038	<0.0001	
BISAP score	0.820	0.800 to 0.838	0.0240	0.5016	1	79.45	70.71	13.331	<0.0001	
90-day mortality										0.0038
PNI	0.726	0.703 to 0.747	0.0308	0.3951	39.80	56.04	83.46	7.338	<0.0001	
BISAP score	0.822	0.802 to 0.840	0.0221	0.5039	1	79.12	71.27	14.558	<0.0001	
180-day mortality										0.0048
PNI	0.726	0.704 to 0.748	0.0279	0.3937	39.80	55.45	83.91	8.095	<0.0001	
BISAP score	0.812	0.792 to 0.831	0.0198	0.4904	1	77.27	71.77	15.768	<0.0001	
1-year mortality										0.0904
PNI	0.729	0.707 to 0.751	0.0228	0.3520	42.55	62.09	73.11	10.049	<0.0001	
BISAP score	0.772	0.750 to 0.792	0.0185	0.4020	1	67.97	72.23	14.713	<0.0001	

ROC, receiver operating characteristic curve; AUC, area under curve; CI, confidence interval; SE, standard error; PNI, prognostic nutritional index; BISAP, bedside index of severity in acute pancreatitis.

### 3.4 Kaplan–Meier survival analysis

**Figure 5:**
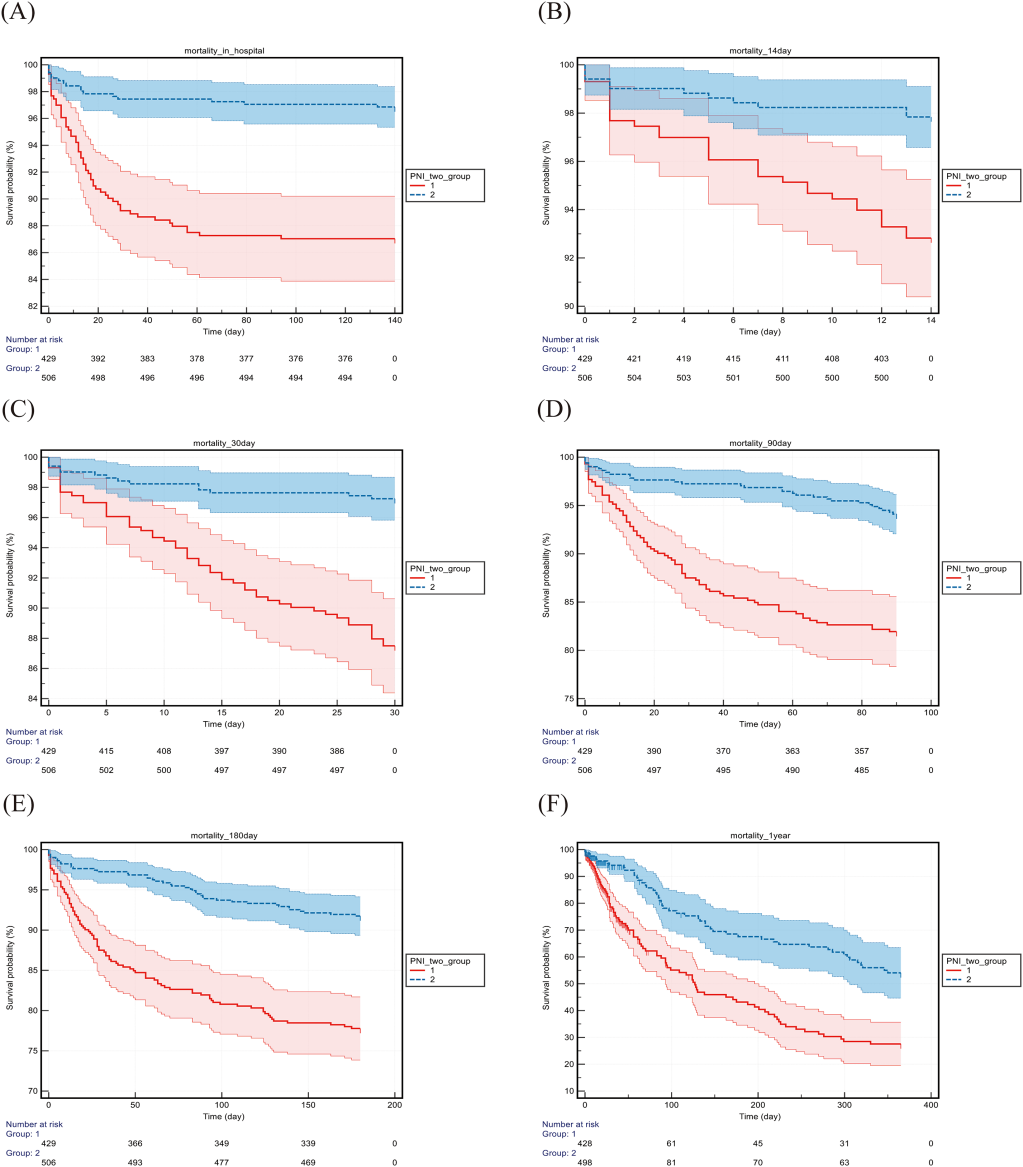
Kaplan–Meier survival analysis from the MIMIC cohort. PNI, prognostic nutritional index; BISAP, bedside index of severity in acute pancreatitis. The horizontal axis represents time in days. The group 1 consists of patients with PNI ≤ 36.66. The group 2 consists of patients with PNI > 36.66.

**Figure 6:**
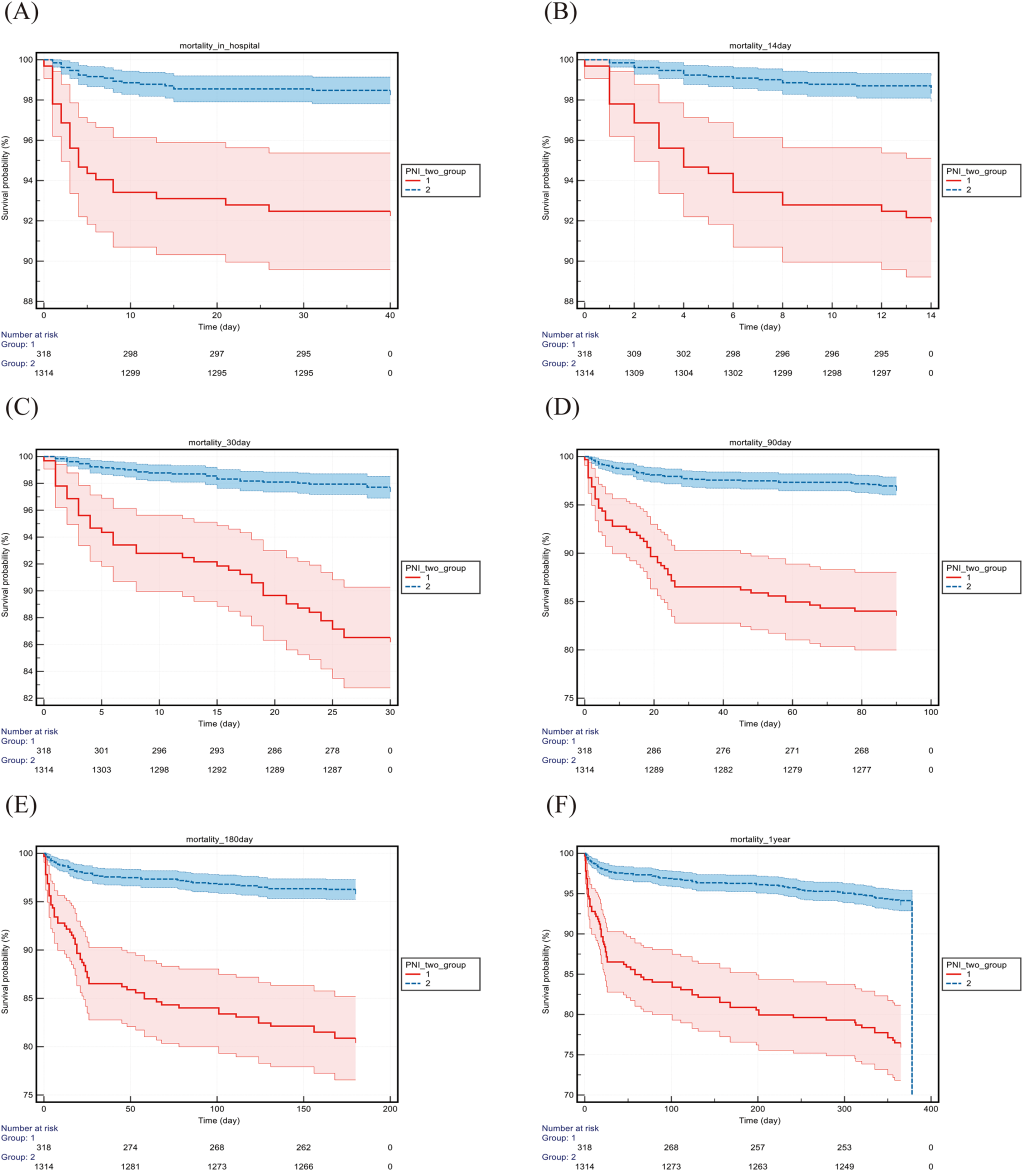
Kaplan–Meier survival analysis from the Chinese cohort. PNI, prognostic nutritional index; BISAP, bedside index of severity in acute pancreatitis. The horizontal axis represents time in days. The group 1 consists of patients with PNI ≤ 40.05. The group 2 consists of patients with PNI > 40.05.

Based on the optimal PNI cutoff values for 30-day mortality rates across the cohorts, patients were divided into low PNI and high PNI groups. Kaplan–Meier survival curves (**Table 4**; **Figures 5**, **6**) showed that survival diverged significantly from admission, with the difference persisting over time. Log-rank tests confirmed that mortality at all time points (hospitalization to 1 year) was significantly higher in the low-PNI group (all P < 0.01). The difference was more pronounced in the Chinese cohort, where low-PNI patients had markedly increased mortality in both the short and long term.

**Table 4:** Kaplan-Meier Survival Analysis of acute pancreatitis by PNI.

Factor		Number of events n (%)	Number censored n (%)	Statistic	Logrank p value
MIMIC	In-hospital mortality			18.7901	< 0.0001
	Group 1	56 (12.96)	376 (87.04)		
	Group 2	16 (3.14)	493 (96.86)		
	14-day mortality			7.4697	0.0063
	Group 1	31 (7.18)	401 (92.82)		
	Group 2	11 (2.16)	498 (97.84)		
	30-day mortality			18.1568	< 0.0001
	Group 1	54 (12.50)	378 (87.50)		
	Group 2	14 (2.75)	495 (97.25)		
	90-day mortality			19.7983	< 0.0001
	Group 1	78 (18.06)	354 (81.94)		
	Group 2	30 (5.89)	479 (94.11)		
	180-day mortality			23.8262	< 0.0001
	Group 1	96 (22.22)	336 (77.78)		
	Group 2	42 (8.25)	467 (91.75)		
	1-year mortality			28.7521	< 0.0001
	Group 1	113 (26.16)	319 (73.84)		
	Group 2	56 (11.00)	453 (89.00)		
CHINA	In-hospital mortality			34.9367	< 0.0001
	Group 1	24 (7.52)	295 (92.48)		
	Group 2	20 (1.52)	1294 (98.48)		
	14-day mortality			39.3371	< 0.0001
	Group 1	25 (7.84)	294 (92.16)		
	Group 2	19 (1.45)	1295 (98.55)		
	30-day mortality			75.0920	< 0.0001
	Group 1	43 (13.48)	276 (86.52)		
	Group 2	30 (2.28)	1284 (97.72)		
	90-day mortality			82.8192	< 0.0001
	Group 1	51 (15.99)	268 (84.01)		
	Group 2	40 (3.04)	1274 (96.96)		
	180-day mortality			99.4445	< 0.0001
	Group 1	61 (19.12)	258 (80.88)		
	Group 2	49 (3.73)	1265 (96.27)		
	1-year mortality			98.7796	< 0.0001
	Group 1	75 (23.51)	244 (76.49)		
	Group 2	78 (5.94)	1236 (94.06)		

PNI, prognostic nutritional index.

### 3.5 Restricted cubic spline analysis of PNI

**Figure 7:**
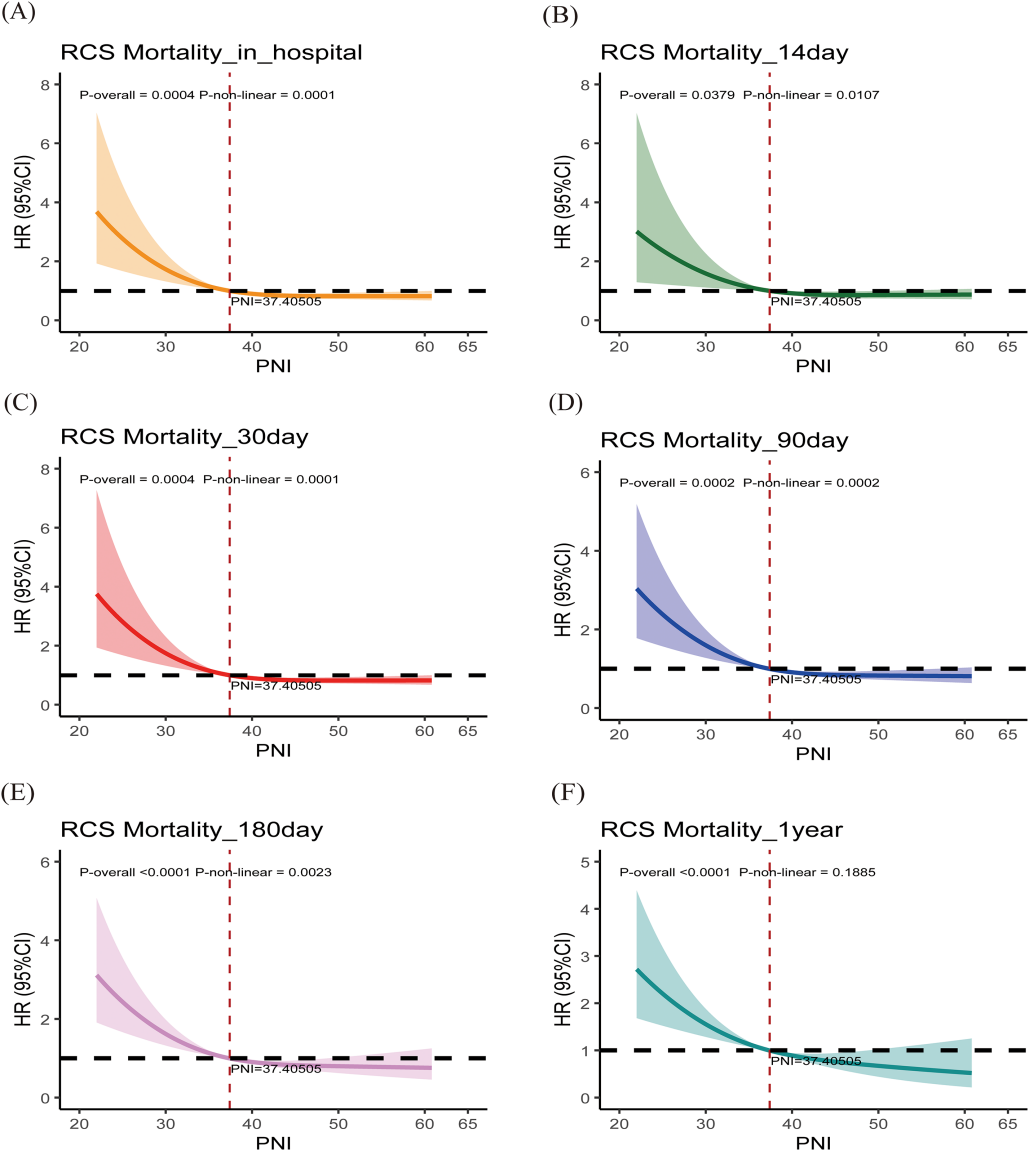
Restricted cubic spline (RCS) analysis from the MIMIC cohort. PNI, prognostic nutritional index. The solid line represents the hazard ratio (HR), and the shaded band represents the 95% confidence interval. The reference line is set at HR = 1. The P-value for overall (P-overall) and P-value for non-linearity (P-non-linear) are displayed on the graph. The X-axis (PNI) is expressed in its original units (dimensionless).

**Figure 8:**
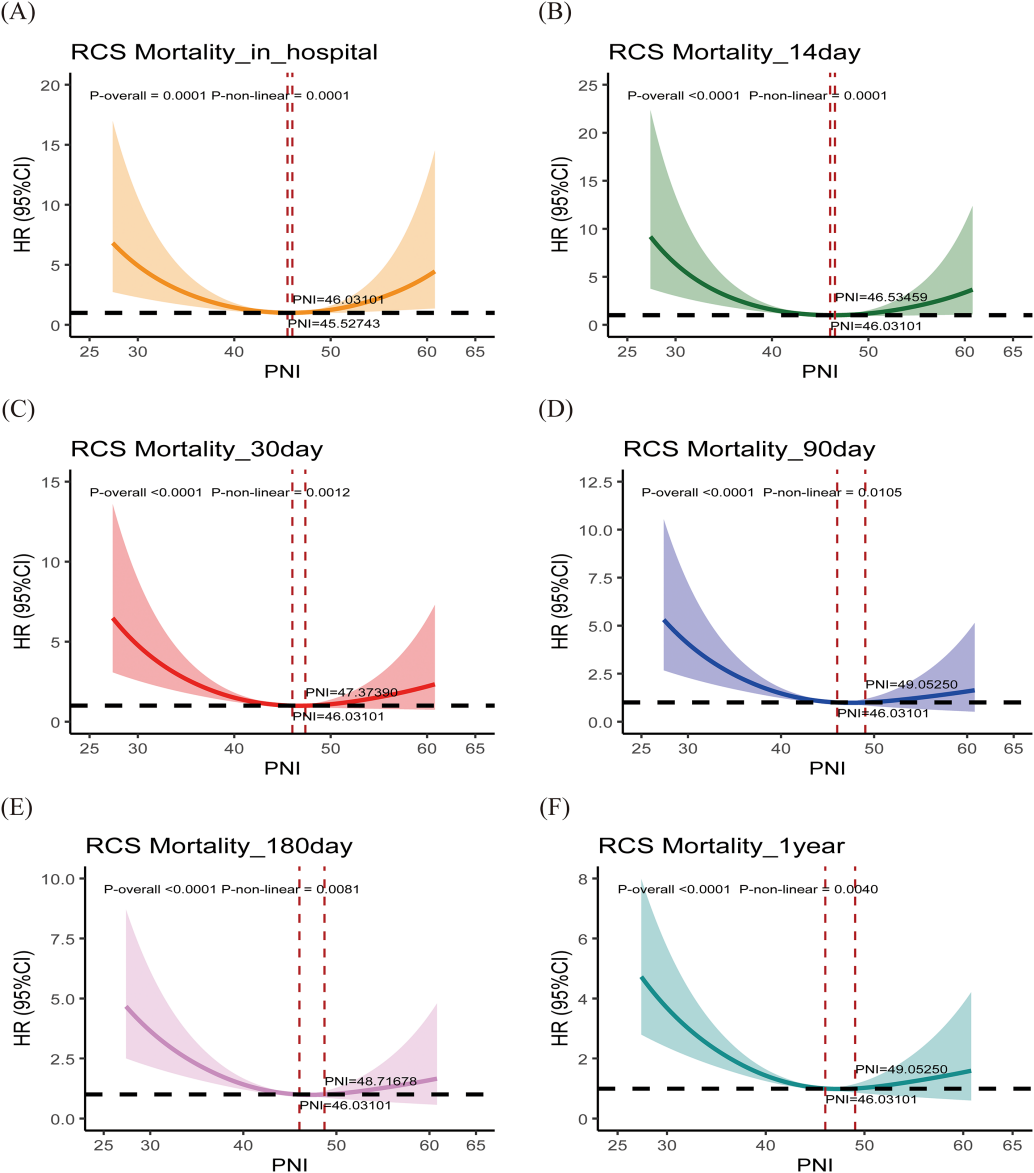
Restricted cubic spline (RCS) analysis from the Chinese cohort. PNI, prognostic nutritional index. The solid line represents the hazard ratio (HR), and the shaded band represents the 95% confidence interval. The reference line is set at HR = 1. The P-value for overall (P-overall) and P-value for non-linearity (P-non-linear) are displayed on the graph. The X-axis (PNI) is expressed in its original units (dimensionless).

After adjusting for age, sex, comorbidities (heart failure, COPD, hypertension, diabetes), and BISAP score, RCS curves (**Figures 7**, **8**) demonstrated that PNI was inversely associated with mortality, with nonlinear effects, except for the MIMIC cohort, where PNI was significantly linearly negatively correlated with 1-year mortality. In the MIMIC cohort, the risk increased sharply below a threshold of ~37 and plateaued above it. In the Chinese cohort, the inflection threshold was higher (~46–49), and low PNI markedly increased both short- and long-term mortality risk.

### 3.6 Subgroup analysis

**Figure 9:**
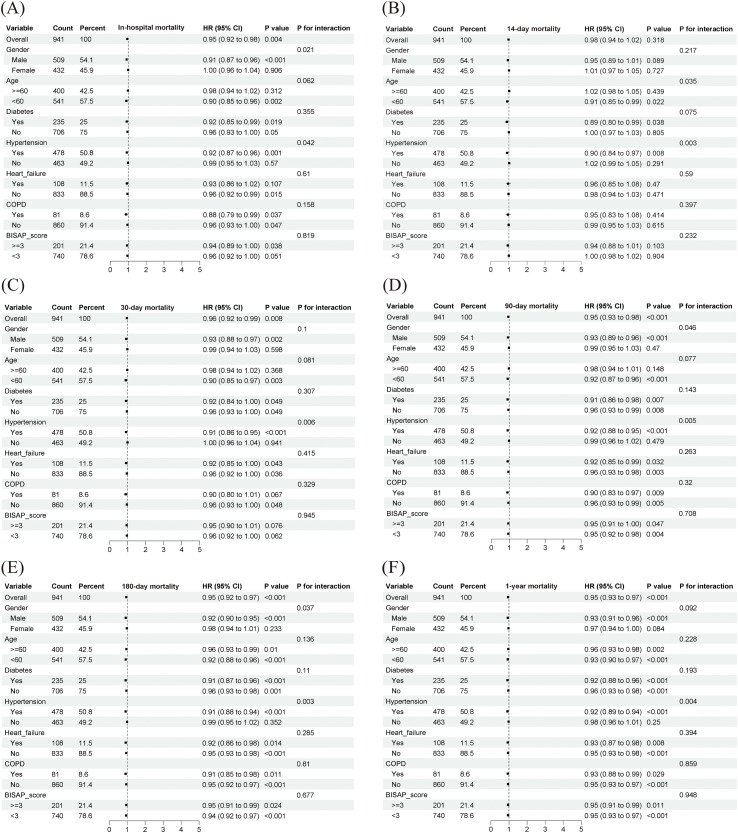
Subgroup analysis from the MIMIC cohort. COPD, chronic obstructive pulmonary disease; BISAP, bedside index of severity in acute pancreatitis. Subgroups are defined by the following criteria: Age (<60 vs. ≥60 years), Gender (Male vs. Female), and BISAP score (<3 vs. ≥3 points). Comorbidities (Hypertension, Diabetes, Heart Failure, COPD) are defined by their presence (Yes) or absence (No). The Hazard Ratio (HR) and 95% Confidence Interval (CI) for each subgroup are presented. The P-value for interaction (P-interaction) is provided to test the difference in effect across subgroups.

**Figure 10:**
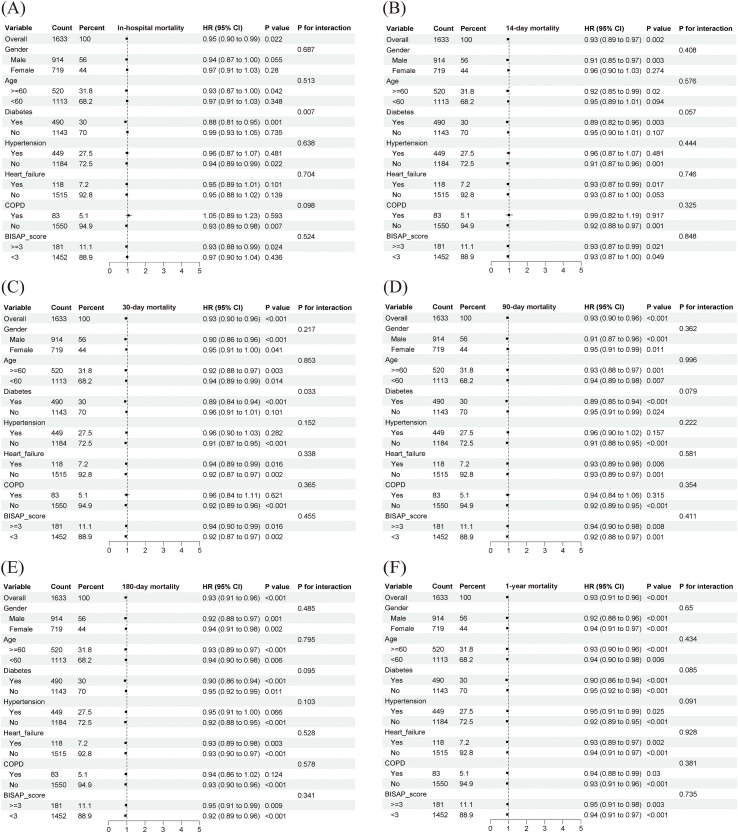
Subgroup analysis from the Chinese cohort. COPD, chronic obstructive pulmonary disease; BISAP, bedside index of severity in acute pancreatitis. Subgroups are defined by the following criteria: Age (<60 vs. ≥60 years), Gender (Male vs. Female), and BISAP score (<3 vs. ≥3 points). Comorbidities (Hypertension, Diabetes, Heart Failure, COPD) are defined by their presence (Yes) or absence (No). The Hazard Ratio (HR) and 95% Confidence Interval (CI) for each subgroup are presented. The P-value for interaction (P-interaction) is provided to test the difference in effect across subgroups.

Subgroup analysis (**Figures 9**, **10**) indicated that the protective association of PNI (HR < 1) was consistent across most strata, suggesting the broad applicability of its association with mortality. Significant interactions were observed (P for interaction < 0.05): in the MIMIC cohort, PNI conferred greater protection in men and in patients with hypertension; in the Chinese cohort, the protective effect was stronger in patients with diabetes.

### 3.7 Mediation analysis of SIRS

**Figure 11:**
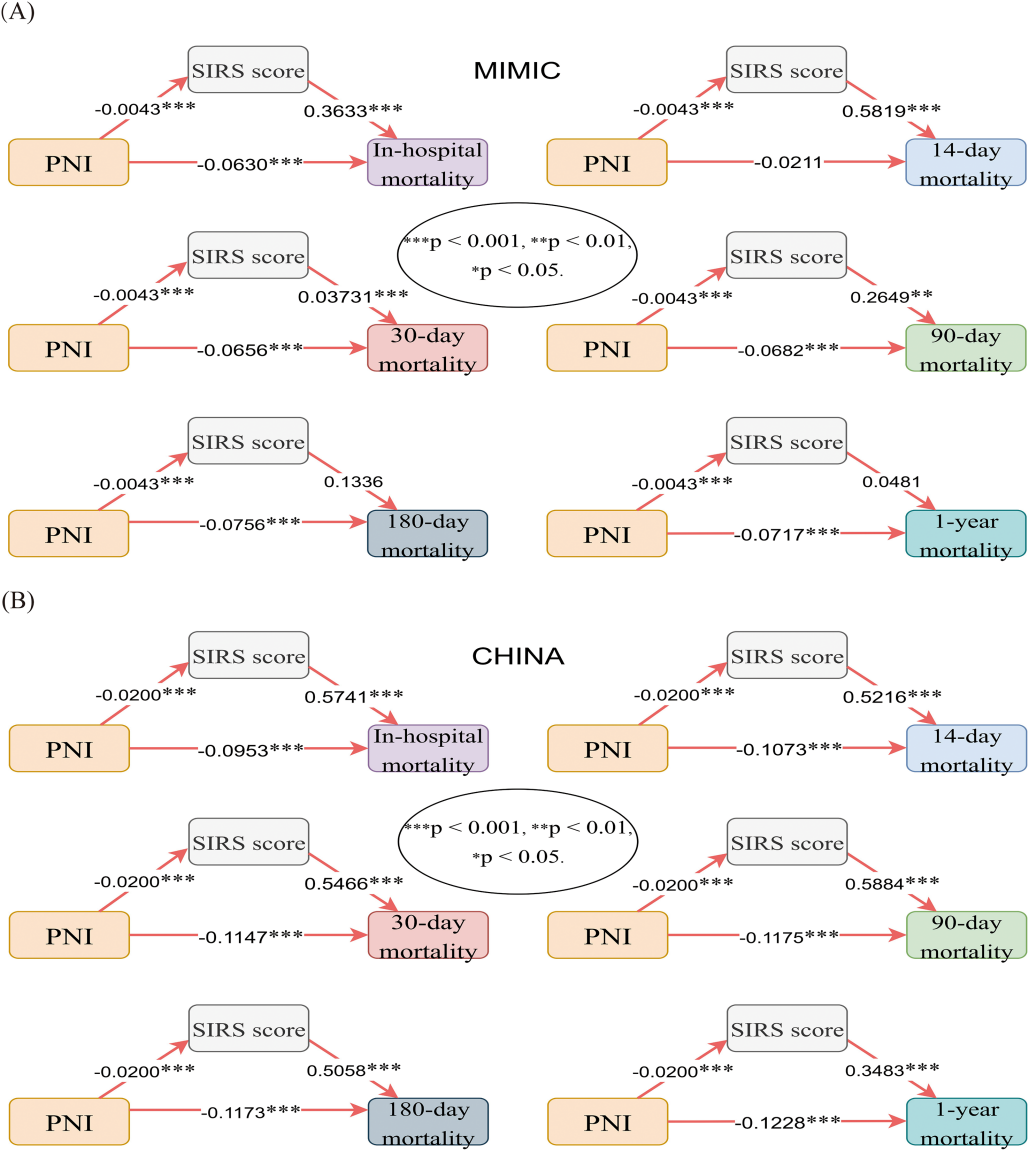
Mediation analysis from the MIMIC cohort and the Chinese cohort. PNI, prognostic nutritional index; SIRS, systemic inflammatory response syndrome. ***, p < 0.001; **, p < 0.01; *, p < 0.05.

Mediation analysis (**Supplementary Table S2**; **Figure 11**) showed that the effect of PNI on mortality was explained in part by the presence of SIRS, although the magnitude differed between cohorts. In the Chinese cohort, mediation was significant at all time points, accounting for 5.39%–10.77% of the total effect. In the MIMIC cohort, mediation was significant only for short-term endpoints (in-hospital, 30-day, 90-day mortality), accounting for 1.59%–2.38% of the total effect.

## 4 Discussion

### 4.1 Principal findings

In this large, international, retrospective cohort study, we demonstrate that the PNI is a robust and independent predictor of both short- and long-term mortality in patients with AP. Two key mechanistic and clinical insights emerged: first, we revealed for the first time that SIRS partially mediates this association, although it is not the primary pathway, providing empirical support for the “nutrition-immunity-inflammation” axis in AP pathogenesis (28–33). Second, the protective effect of PNI exhibits a nonlinear threshold relationship, with mortality risk escalating sharply below cohort-specific inflection points. Importantly, PNI offered complementary prognostic value to the established BISAP score, particularly for long-term outcomes, suggesting its potential utility in comprehensive risk stratification.

### 4.2 Interpretation and mechanistic insights

The central role of PNI, a composite marker of nutritional status (albumin) and immune competence (lymphocytes) (28, 29), underscores the critical interplay between host constitution and disease outcomes in AP. Our mediation analysis offers a novel pathophysiological explanation for this link. We found that SIRS statistically accounted for a portion of PNI’s effect on mortality. This suggests that a lower PNI may predispose patients to a more severe or sustained systemic inflammatory state, which in turn increases the risk of organ failure and death (30–33).

The biological mechanisms underlying this association are complex and multifaceted. Lymphocytopenia and hypoalbuminemia, reflected by a low PNI, collectively foster an immune environment prone to dysregulation (32–35). Lymphocytes, particularly T cells, are crucial for coordinating appropriate immune responses, preventing excessive immune activation, and facilitating tissue repair (36, 37). Lymphocytopenia impairs cellular immunity, weakens control of secondary infections, and may contribute to an imbalance between pro-inflammatory and anti-inflammatory signaling, such as the well-documented increase in the IL-6 (pro-inflammatory) to IL-10 (anti-inflammatory) ratio, which is strongly associated with poor outcomes in AP (38–40). Thus, a low PNI may signal exhausted immune compensatory capacity, leaving the patient unable to effectively constrain the initial inflammatory storm, leading to the persistence and worsening of SIRS (31–33, 36).

Concurrently, the role of serum albumin extends far beyond that of a nutritional marker (41). Beyond its classic roles in maintaining intravascular volume and endothelial function, albumin plays a key part in sustaining antioxidant defenses and immune homeostasis (41, 42). As the primary antioxidant in blood, albumin scavenges reactive oxygen species via its thiol groups, mitigating the oxidative stress characteristic of AP (43, 44). Hypoalbuminemia compromises this protection, exacerbating tissue damage. Furthermore, albumin binds and neutralizes various endogenous and exogenous toxins and modulates leukocyte activation and cytokine signaling (43, 45, 46). Therefore, low albumin levels not only reflect disease severity but also actively participate in the process of immune dysregulation by exacerbating oxidative damage and altering immune cell communication, providing a substrate for the perpetuation of SIRS (41–43).

Nevertheless, the predominance of PNI’s direct effect indicates that other mechanisms are paramount. These may include the non-immunological functions of albumin mentioned above and the direct role of lymphocytes in tissue repair. The weaker mediation effect of SIRS in the more critically ill MIMIC ICU cohort may reflect that in these patients, non-inflammatory pathways of organ injury (e.g., ischemic or toxic) become dominant.

### 4.3 Clinical implications and translation

Our results provide a compelling argument for integrating the PNI into the initial clinical assessment of AP patients. To translate these findings into practice, we propose a structured approach for risk stratification and targeted intervention. Based on our analyses, population-specific PNI thresholds can identify high-risk patients: a value below 37 in populations similar to the MIMIC cohort, and below 46 in Chinese cohort, signifies significant nutritional and immunologic compromise. Identifying these high-risk patients should trigger a proactive, bundled management strategy. This strategy must include protocolized nutritional support that moves beyond simple caloric replenishment. The focus should be on strategies that support endogenous albumin synthesis and lymphocyte function, making a strong case for the early introduction of immunonutrition (47, 48). Formulas enriched with substrates like glutamine, arginine, and omega-3 fatty acids may directly counter the immune dysfunction characteristic of a low PNI state (47, 48). The complementary strengths of BISAP for short-term severity and PNI for long-term risk argue for their combined use in a practical clinical algorithm. We propose that all AP patients undergo dual assessment at admission. Those with a high BISAP score (≥3) require intensive care irrespective of PNI. However, patients with a low BISAP score (<3) but a low PNI constitute a critical, high-risk subgroup vulnerable to under-triaging. These patients should receive enhanced monitoring on the general ward and be prioritized for early, protocolized nutritional support, including immunonutrition, alongside vigilant monitoring for SIRS. This integrated model ensures a more personalized and proactive management strategy, directing resources effectively to improve outcomes across the risk spectrum.

### 4.4 Generalizability and population specificity

Our findings that PNI is an independent protective factor are consistent with a growing body of evidence on nutrition-inflammation markers in critical care (49, 50). The nonlinear relationship revealed by RCS analysis is a significant advancement, indicating that the mortality risk is not linear but accelerates markedly below a critical threshold. The higher inflection point in the Chinese cohort (~46) compared to the MIMIC cohort (~37) likely reflects differences in baseline population health and disease severity, underscoring the need for population-specific benchmarks. Furthermore, the consistent protective effect of PNI across most subgroups (sex, age, comorbidities) strengthens the generalizability and reliability of our conclusions. Our international cohort design intentionally incorporated heterogeneity to test the robustness of the PNI-mortality association. The observed differences in baseline characteristics, optimal PNI cut-off values, and the magnitude of SIRS mediation between the MIMIC and Chinese cohorts underscore the population-specific characteristics of each healthcare setting. Importantly, the consistent, independent, and graded association between PNI and mortality across these diverse contexts strongly supports the external validity and general prognostic value of PNI in acute pancreatitis. However, these differences also indicate that the specific operational thresholds for risk stratification and the relative importance of inflammatory mediation may be context-dependent. Therefore, while the prognostic utility of PNI appears generalizable, clinical implementation strategies might benefit from local validation of cut-off values.

### 4.5 Limitations

This study has several limitations that should be considered when interpreting the results. First, the retrospective and observational nature of our design precludes definitive causal inference, and residual confounding cannot be fully excluded. Although we adjusted for a core set of robust and consistently available confounders (demographics, key comorbidities, and the BISAP score), the choice of variables was necessarily constrained by the international retrospective design. To ensure data quality and comparability across the U.S. and Chinese cohorts, we prioritized well-defined, universally collected prognostic factors over a larger set of variables with potential heterogeneity or significant missingness. Consequently, we were unable to adjust for other potential confounders such as the detailed etiology of AP, specific nutritional interventions, fluid management strategies, or socioeconomic factors, which might influence the outcomes. The mediation analysis also relies on strong assumptions regarding unmeasured confounding across the PNI–SIRS–mortality pathways. To enhance robustness, we ensured temporal precedence of PNI assessment, adjusted for multiple confounders including BISAP score, verified model assumptions (proportional hazards, no PNI–SIRS interaction), and used bootstrap methods for indirect effect estimation.

Second, the assessment timing of our key variables differed. PNI and BISAP were calculated from baseline data within 24 hours of admission, providing a valuable early snapshot but not capturing their potential dynamic changes throughout the hospitalization. Conversely, while SIRS was assessed dynamically to establish its role as a mediator occurring after the baseline PNI measurement, this also means that the SIRS status we used (the highest score during hospitalization) does not strictly represent a single, fixed post-baseline time point. This approach, while methodologically sound for establishing the occurrence of SIRS, does not allow us to model the precise temporal interplay or the longitudinal trajectories of these variables. Serial measurements of PNI, SIRS, and other parameters would provide a more comprehensive pathophysiological perspective on how the evolution of nutritional and inflammatory status impacts prognosis.

Third, severity was assessed using the BISAP score due to its practical advantages in our retrospective multinational design - specifically, its reliance on objectively extractable parameters available across both cohorts. Although the Revised Atlanta Classification remains the gold standard for phenotyping, BISAP is a validated predictor of our primary outcomes (mortality and organ failure). After rigorous adjustment for BISAP in all models, the consistent PNI-mortality association across subgroups supports its independence from underlying disease severity. Fourth, inter-laboratory methodological variations between the U.S. and Chinese centers may introduce measurement bias and affect the absolute comparability of laboratory values used to calculate PNI and SIRS. However, the clinical interpretation of these values (e.g., hypoalbuminemia, leukocytosis) is standardized, and the consistent prognostic performance of PNI across both cohorts suggests that such potential bias did not substantially alter the primary conclusions. What’s more, we employed a complete-case analysis for handling missing data. While the missingness was limited, this approach assumes data are missing completely at random and may reduce statistical power.

Finally, it is important to note that SIRS served only as a partial mediator, explaining a relatively small proportion of the total effect of PNI on mortality. This key finding indicates that the protective effect of a high PNI is not primarily mediated through the attenuation of early systemic inflammation and underscores the significant contribution of other biological pathways. These likely include, but are not limited to, the preservation of intestinal barrier function, modulation of the gut microbiome, and fine-tuning of the cytokine network, all of which warrant explicit investigation in future studies. Furthermore, as an observational study, our work establishes a strong association but cannot prove causality. Future studies should prospectively track nutritional and inflammatory markers over time and systematically collect data on nutritional and treatment details. Interventional trials should assess whether improving PNI directly reduces inflammation and mortality in AP patients.

## 5 Conclusion

This study demonstrated, using two large international cohorts with inherent clinical and methodological heterogeneity, that PNI is an independent predictor of mortality in acute pancreatitis. The association was robust across both cohorts, supporting its broad prognostic utility. We revealed for the first time that SIRS is a significant but partial mediator, indicating the involvement of additional mechanisms. The generalizability of our findings is enhanced by the consistency of the association across diverse populations, although the specific PNI thresholds and mediating pathways may exhibit population-specific characteristics. Consequently, PNI should be considered for integration into risk stratification systems, with the understanding that local calibration may be optimal. Future research should focus on prospective intervention and on elucidating the other biological pathways linking nutritional status to survival.

## 6 Ethical approval

The establishment of the MIMIC-IV database was approved by the Institutional Review Board of the Massachusetts Institute of Technology (Cambridge, Massachusetts) and Beth Israel Deaconess Medical Center (Boston, Massachusetts), and consent for the original data collection was obtained. Therefore, the requirement for ethical approval statements and informed consent in this manuscript is waived. This retrospective study was approved by the Medical Ethics Committee of the Affiliated Yongchuan Hospital of Chongqing Medical University for clinical/scientific projects, registration number 2025EC0030. This study employed a retrospective approach, with data extracted from the database of the Information Center at the Affiliated Yongchuan Hospital of Chongqing Medical University. De-identification of privacy and personal identifying information was performed for research subjects. As the study did not involve destructive or sensitive research on subjects, the Medical Ethics Committee of the Affiliated Yongchuan Hospital of Chongqing Medical University granted exemption from informed consent.

## Data Availability

Publicly available datasets were analyzed in this study. This data can be found here: The clinical data used in this study were obtained from the publicly available Medical Information Mart for Intensive Care (MIMIC) databases. MIMIC data can be accessed via PhysioNet upon completion of the required data use training and credentialing. MIMIC-IV (v2.2): Repository: PhysioNet. Direct link: https://physionet.org/content/mimiciv/. The Chinese cohort dataset is not publicly available due to institutional policies but can be provided by the corresponding author upon reasonable request and with approval from the appropriate institutional review boards.
