# Supplementary material for: Inflammation mediates the association between the Prognosis Nutrition Index (PNI) and mortality in acute pancreatitis: evidence from international cohorts
Source: Front Immunol. 2026 Jan 14;16:1693888. doi: 10.3389/fimmu.2025.1693888 (PMC12846994; doi:10.3389/fimmu.2025.1693888)
Supplement: Supplementary file 1 [file Table1.docx]

# **Supporting materials**

Table S1 Shared Frailty Cox Model

| Categories | Model 1  HR(95%CI) | Model 2  HR(95%CI) | Model 3  HR(95%CI) |
| --- | --- | --- | --- |
| In-hospital mortality (PNI) | 0.923(0.899,0.948) | 0.928(0.904,0.954) | 0.956(0.930,0.983) |
| 14-day mortality (PNI) | 0.925(0.902,0.948) | 0.930(0.907,0.955) | 0.957(0.931,0.984) |
| 30-day mortality (PNI) | 0.909(0.891,0.926) | 0.914(0.896,0.933) | 0.942(0.922,0.963) |
| 90-day mortality (PNI) | 0.917(0.898,0.935) | 0.922(0.903,0.941) | 0.949(0.928,0.969) |
| 180-day mortality (PNI) | 0.919(0.902,0.935) | 0.926(0.909,0.943) | 0.946(0.929,0.965) |
| 1-year mortality (PNI) | 0.920(0.905,0.934) | 0.929(0.914,0.944) | 0.945(0.929,0.960) |

Note: COX, proportional hazards model; HR, hazard ratio; CI, confidence interval; PNI, prognostic nutritional index; COPD, chronic obstructive pulmonary disease; BISAP, bedside index of severity in acute pancreatitis;

Model 1: No confounding factors were adjusted;

Model 2: Incorporated adjustments for Age and Gender;

Model 3: Incorporated adjustments for Age, Gender, COPD, Heart failure, Diabetes, Hypertension and BISAP score.

Table S2 Intermediary analytics

| Factor | Efficiency value | SE | LLCI | ULCI | Effect size (%) |
| --- | --- | --- | --- | --- | --- |
| **MIMIC** |  |  |  |  |  |
| PNI—SIRS score—In-hospital mortality |  |  |  |  |  |
| Direct effect | -0.0630 | 0.0187 | -0.0997 | -0.0264 | 97.67 |
| Intermediary effect | -0.0015 | 0.0065 | -0.0233 | -0.0005 | 2.33 |
| PNI—SIRS score—14-day mortality |  |  |  |  |  |
| Direct effect | -0.0211 | 0.0227 | -0.0656 | 0.0234 | 0.00 |
| Intermediary effect | -0.0025 | 0.0098 | -0.0358 | -0.0009 | 100.00 |
| PNI—SIRS score—30-day mortality |  |  |  |  |  |
| Direct effect | -0.0656 | 0.0193 | -0.1035 | -0.0277 | 97.62 |
| Intermediary effect | -0.0016 | 0.0066 | -0.0243 | -0.0005 | 2.38 |
| PNI—SIRS score—90-day mortality |  |  |  |  |  |
| Direct effect | -0.0682 | 0.0155 | -0.0986 | -0.0378 | 98.41 |
| Intermediary effect | -0.0011 | 0.0048 | -0.0170 | -0.0003 | 1.59 |
| PNI—SIRS score—180-day mortality |  |  |  |  |  |
| Direct effect | -0.0756 | 0.0139 | -0.1029 | -0.0483 | 100.00 |
| Intermediary effect | -0.0006 | 0.0027 | -0.0099 | 0.0002 | 0.00 |
| PNI—SIRS score—1-year mortality |  |  |  |  |  |
| Direct effect | -0.0717 | 0.0126 | -0.0965 | -0.0470 | 100.00 |
| Intermediary effect | -0.0002 | 0.0019 | -0.0061 | 0.0022 | 0.00 |
| **CHINA** |  |  |  |  |  |
| PNI—SIRS score—In-hospital mortality |  |  |  |  |  |
| Direct effect | -0.0953 | 0.0227 | -0.1397 | -0.0508 | 89.23 |
| Intermediary effect | -0.0115 | 0.0041 | -0.0212 | -0.0051 | 10.77 |
| PNI—SIRS score—14-day mortality |  |  |  |  |  |
| Direct effect | -0.1073 | 0.0227 | -0.1518 | -0.0629 | 91.16 |
| Intermediary effect | -0.0104 | 0.0038 | -0.0192 | -0.0042 | 8.84 |
| PNI—SIRS score—30-day mortality |  |  |  |  |  |
| Direct effect | -0.1147 | 0.0184 | -0.1507 | -0.0787 | 91.32 |
| Intermediary effect | -0.0109 | 0.0035 | -0.0190 | -0.0049 | 8.68 |
| PNI—SIRS score—90-day mortality |  |  |  |  |  |
| Direct effect | -0.1175 | 0.0169 | -0.1506 | -0.0843 | 90.87 |
| Intermediary effect | -0.0118 | 0.0036 | -0.0199 | -0.0056 | 9.13 |
| PNI—SIRS score—180-day mortality |  |  |  |  |  |
| Direct effect | -0.1173 | 0.0156 | -0.1478 | -0.0868 | 92.07 |
| Intermediary effect | -0.0101 | 0.0031 | -0.0168 | -0.0048 | 7.93 |
| PNI—SIRS score—1-year mortality |  |  |  |  |  |
| Direct effect | -0.1228 | 0.0137 | -0.1496 | -0.0959 | 94.61 |
| Intermediary effect | -0.0070 | 0.0023 | -0.0120 | -0.0032 | 5.39 |

Note: PNI, prognostic nutritional index; SIRS, systemic inflammatory response syndrome; SE, standard error; LLCI, lower level of confidence interval; ULCI, upper level of confidence interval;

Because the outcome variable (presence or absence of mortality) is dichotomous, total effect sizes could not be calculated directly.
